# The Ser82 RAGE Variant Affects Lung Function and Serum RAGE in Smokers and sRAGE Production *In Vitro*

**DOI:** 10.1371/journal.pone.0164041

**Published:** 2016-10-18

**Authors:** Suzanne Miller, Amanda P. Henry, Emily Hodge, Alexander K. Kheirallah, Charlotte K. Billington, Tracy L. Rimington, Sangita K. Bhaker, Ma’en Obeidat, Erik Melén, Simon K. Merid, Caroline Swan, Catherine Gowland, Carl P. Nelson, Ceri E. Stewart, Charlotte E. Bolton, Iain Kilty, Anders Malarstig, Stuart G. Parker, Miriam F. Moffatt, Andrew J. Wardlaw, Ian P. Hall, Ian Sayers

**Affiliations:** 1 Division of Respiratory Medicine, University of Nottingham, Nottingham, United Kingdom; 2 Institute of Environmental Medicine, Karolinska Institutet, Stockholm, Sweden; 3 Department of Biology, University of York, York, United Kingdom; 4 Pfizer Worldwide Research & Development, Cambridge, Massachusetts, United States of America; 5 Pfizer Worldwide Research & Development, Cambridge, United Kingdom; 6 Institute for Ageing and Health, Newcastle University, Campus for Ageing and Vitality, Newcastle upon Tyne, United Kingdom; 7 National Heart and Lung Institute, Imperial College London, London, United Kingdom; 8 Institute for Lung Health, Immunity and Inflammation, University of Leicester, Leicester, United Kingdom; University of Pittsburgh, UNITED STATES

## Abstract

**Introduction:**

Genome-Wide Association Studies have identified associations between lung function measures and Chronic Obstructive Pulmonary Disease (COPD) and chromosome region 6p21 containing the gene for the Advanced Glycation End Product Receptor (*AGER*, encoding RAGE). We aimed to (i) characterise RAGE expression in the lung, (ii) identify *AGER* transcripts, (iii) ascertain if SNP rs2070600 (Gly82Ser C/T) is associated with lung function and serum sRAGE levels and (iv) identify whether the Gly82Ser variant is functionally important in altering sRAGE levels in an airway epithelial cell model.

**Methods:**

Immunohistochemistry was used to identify RAGE protein expression in 26 human tissues and qPCR was used to quantify *AGER* mRNA in lung cells. Gene expression array data was used to identify *AGER* expression during lung development in 38 fetal lung samples. RNA-Seq was used to identify *AGER* transcripts in lung cells. sRAGE levels were assessed in cells and patient serum by ELISA. BEAS2B-R1 cells were transfected to overexpress RAGE protein with either the Gly82 or Ser82 variant and sRAGE levels identified.

**Results:**

Immunohistochemical assessment of 6 adult lung samples identified high RAGE expression in the alveoli of healthy adults and individuals with COPD. *AGER*/RAGE expression increased across developmental stages in human fetal lung at both the mRNA (38 samples) and protein levels (20 samples). Extensive *AGER* splicing was identified. The rs2070600T (Ser82) allele is associated with higher FEV_1,_ FEV_1_/FVC and lower serum sRAGE levels in UK smokers. Using an airway epithelium model overexpressing the Gly82 or Ser82 variants we found that HMGB1 activation of the RAGE-Ser82 receptor results in lower sRAGE production.

**Conclusions:**

This study provides new information regarding the expression profile and potential role of RAGE in the human lung and shows a functional role of the Gly82Ser variant. These findings advance our understanding of the potential mechanisms underlying COPD particularly for carriers of this *AGER* polymorphism.

## Introduction

Lung diseases including chronic obstructive pulmonary disease (COPD) remain a major burden to society and the economy. In recent years there has been substantial progress in our understanding of genetic factors associated with lung function parameters through genome wide association approaches [1–4]. There is a key need however, to define the mechanisms which underlie the identified associations. Meta-analyses of genome-wide association studies (GWAS) have identified polymorphisms in the Advanced Glycation End Product-Specific gene (*AGER*, pivotal SNP in exon 3 rs2070600 (Gly82Ser, C/T)) as being associated with Forced Expiratory Volume in 1 second (FEV_1_)/Forced Vital capacity (FVC) [1, 2]. The role of *AGER* now requires further elucidation in the lung which may provide new insight into the mechanisms underlying several lung diseases where *AGER* genetic variants have been implicated, including e.g. COPD and lung cancer [5, 6]. *AGER* encodes RAGE; a member of the immunoglobulin superfamily which interacts with a diverse range of ligands, including damage associated molecular pattern proteins such as High mobility group protein B1 (HMGB1). Activation of RAGE has many downstream effects including the regulation of inflammation, cell migration, proliferation and adhesion [7, 8]. Importantly, accumulating data suggests RAGE may play a role in a range of diseases including COPD [5, 9, 10], Alzheimer’s disease, arthritis and diabetes [11–13] and so a greater understanding of *AGER* regulation by genetic mechanisms has broad implications. RAGE is expressed abundantly in the membrane and cytoplasm of both pneumocytes and macrophages in lung [14], suggesting an important role in lung homeostasis. Recent studies in transgenic mouse models have shown a role for RAGE in alveolar morphogenesis during lung development [15] with overexpression of RAGE also being shown to result in the development of an emphysema-like phenotype in adult mice [16]. Increased expression of RAGE, or the soluble forms of the receptor generated by cleavage (sRAGE) or mRNA splicing (esRAGE), has been found in the airways and serum of COPD patients [5, 9, 10]. However, the mechanism underlying the genetic association with lung function seen at this locus has as yet not been defined. In the current study we set out specifically to further define the potential role of *AGER* in the context of respiratory disease. To do this we have determined the expression profile of RAGE in human lung and during lung development. Additionally, we have investigated the specific functional effects of the key Gly82Ser variant that has been associated with numerous lung phenotypes at both the population and cellular levels providing an insight into the function of this variant in the lung.

## Methods

### Immunohistochemistry

Three control (no history of obstructive lung disease) adult lung samples and three samples from individuals with clinically diagnosed COPD were provided by the Nottingham Health Science Biobank following isolation by lung resection (Nottingham, UK) under ethical approval (08/H0407/1). Twenty fetal lung tissue samples were provided by The Human Developmental Biology Resource (www.hdbr.org) at different stages, specifically 19, 21 and 23 days and 9, 10, 11, 12, 13, 14, 15, 16, 17 and 19 weeks post-conception. Immunohistochemistry was performed as previously described [17]. Slides were incubated with either a rabbit polyclonal anti-RAGE antibody (1:500, ab30381, Abcam, Cambridge, UK) or normal rabbit IgG isotype control (Invitrogen/Life Technologies Ltd.) for 1 hour at room temperature.

### Affymetrix U133 Plus 2 array data for human fetal lung

Prior studies have obtained human fetal lung tissues from the National Institute of Child Health and Human Development tissue databases and the extracted RNA profiled using microarrays to investigate gene expression spanning different gestational ages [18, 19]. RNA samples from 38 subjects (estimated gestational age 7–22 weeks or 53–154 days post conception) *i*.*e*. Pseudoglandular (gestational age, 7–16 weeks) and into the Canalicular (17–26 weeks) stages of development were used in the current *AGER* analyses. These data are available at NCBI Gene Expression Omnibus (GEO, http://www.ncbi.nlm.nih.gov/geo), GSE14334. Affymetrix probes; 217046_s_at and 210081_at were used for *AGER* expression analyses in relation to gestational age (linear regression model (lmFit) as implemented in the Limma package in R/Bioconductor).

### Quantitative PCR

Quantification of total *AGER* mRNA was carried out using total RNA from cultured cells or commercially available total lung tissue (Ambion/Life Technologies) using Taqman^™^ (Applied Biosystems/Life Technologies) as previously described [20]. In each sample, quantitative PCR (qPCR) was carried out for *AGER* (Assay VY4331182 designed to detect part of the 5’region and thus detects the majority of transcripts, Invitrogen) and Human 18S ribosomal RNA 1 endogenous control (4310893E, Applied Biosystems). qPCR was performed using a Stratagene Mx3005P machine (Agilent Technologies, UK). Data were analysed using the 2^-ΔΔCt^ method.

### RNA Sequencing

Total RNA was extracted from normal human bronchial epithelial cells (NHBECs) from three donors (Lonza, UK) at either passage 3 or 4 using the GenElute Mammalian Total RNA Miniprep Kit (Sigma-Aldrich). Donor 1 was a Caucasian, 56 years old, Male, Smoker; Donor 2 was a 19 year old, Male, Smoker and Donor 3 was a Caucasian, 50 year old, Male, Smoker. RNA quality was assessed using the Agilent 2100 Bioanalyzer with all samples having a RNA integrity number ≥ 8. A sequencing library was prepared using TruSeq RNA Sample Prep Kit v2 (Illumina). mRNA was poly-A selected using oligo-dT coated magnetic beads and fragmented. cDNA was synthesised using random primers. Finally, a cDNA library was prepared by end-repair, phosphorylation, A-tailing, adapter ligation and PCR amplification. Paired-end sequencing was performed on either the Illumina HiSeq 2000 or the NextSeq 500 with approximately 40 million reads per sample generated. RNA-seq data was analysed as described [21]. The raw read FastQ files (100 base pair reads) were quality evaluated using FastQC. Median quality scores were above 28 for all samples. Reads were used for subsequent analysis using the Ubuntu 12.04 LTS operating system. Reads were aligned to the human genome (Build GRCh37) for each sample using Bowtie2 tool as part of TopHat (v2.0.12). Reads aligning to more than 20 positions were discarded. The Cufflinks v2.2.1 programme was used to assemble transcriptomes for each individual sample. Transcriptomes from all the samples were merged by reference annotation based transcript assembly (RABTA) using Cuffmerge v1.0.0 to provide the maximum reads to identify low-expression transcripts. The Cuffmerge generated novel gene transfer format (GTF) annotation file was compared to the Ensembl GTF annotation of the GRCh37 genome build using Cuffcompare v2.2.1. Basal NHBEC sample data (n = 3) were used to identify transcript abundance. Mean fragments per kilobase of transcript per million fragments mapped (FPKM) expression of isoforms was calculated and percent of total transcripts determined. Splicing graphs were generated using SpliceGrapher v0.2.4.

### Patient recruitment, genotyping and ELISA

Subjects were recruited from five UK centres for smoking history and/or COPD diagnosis, this cohort is the same as that previously described [22, 23]. This generated a cohort for lung function association analyses composed of 1042 smokers with DNA samples available. rs2070600 was genotyped using the LGC Genomics KBioscience service (Herts, UK). We generated a sub-group based on rs2070600 genotype composed of 102 subjects for serum analyses (51 C:T and 51 C:C age, sex and smoking pack year matched). The level of sRAGE in the serum was determined using the Quantikine ELISA kit for Human RAGE as directed by the manufacturer (R & D systems, UK). Ethical approval was obtained from local ethics committees (Nottingham, Sheffield, Manchester, Leicester and Oxford).

### Site-directed mutagenesis and transfection of BEAS2B-R1 cells

A plasmid containing the cDNA for the full length, membrane form of RAGE (pcDNA3.1-flRAGE) was a kind gift of Dr. B. Hudson (University of Miami). Site directed mutagenesis of the flRAGE plasmid was carried out using QuikChange^®^ Multi Site-directed Mutagenesis kit (Agilent, UK). A single mutagenic primer (CGTGTCCTTCCCAAC**(A)**GCTCCCTCTTCCT) was used to generate the alternative allele of SNP rs2070600. All constructs were sequence verified (data not shown). BEAS2B-R1 epithelial cells (provided by Dr. Ray Penn, University of Maryland, Baltimore, USA) [24] were transiently transfected with plasmids using Fugene 6 (Promega, UK) at a 3:2 Fugene:DNA ratio, grown for 24 hours and then serum starved for 16 hours. 250ng/ml of HMGB1 ligand (R & D systems, UK, 1690-HMB-025) was then added to the cells for 48 hours. Supernatants were collected for analysis and sRAGE levels measured as described for serum analyses. Cells were lysed and RNA extracted using the GenElute Mammalian Total RNA miniprep kit (Sigma). cDNA was synthesised using the Superscript First-Strand Synthesis System for RT-PCR (Invitrogen/Life Technologies). qPCR for *AGER* was performed as described. Data from four independent cell experiments were combined for analyses.

### Statistical analysis

Linear regression was used to assess association between lung function measures (FEV_1_, FVC and FEV_1_/FVC) and rs2070600 genotype or log serum sRAGE levels as previously described [2]. Mann-Whitney tests were used to compare groups (GraphPad Prism 6).

## Results

### RAGE expression and localisation in healthy and COPD lungs

**Fig 1:**
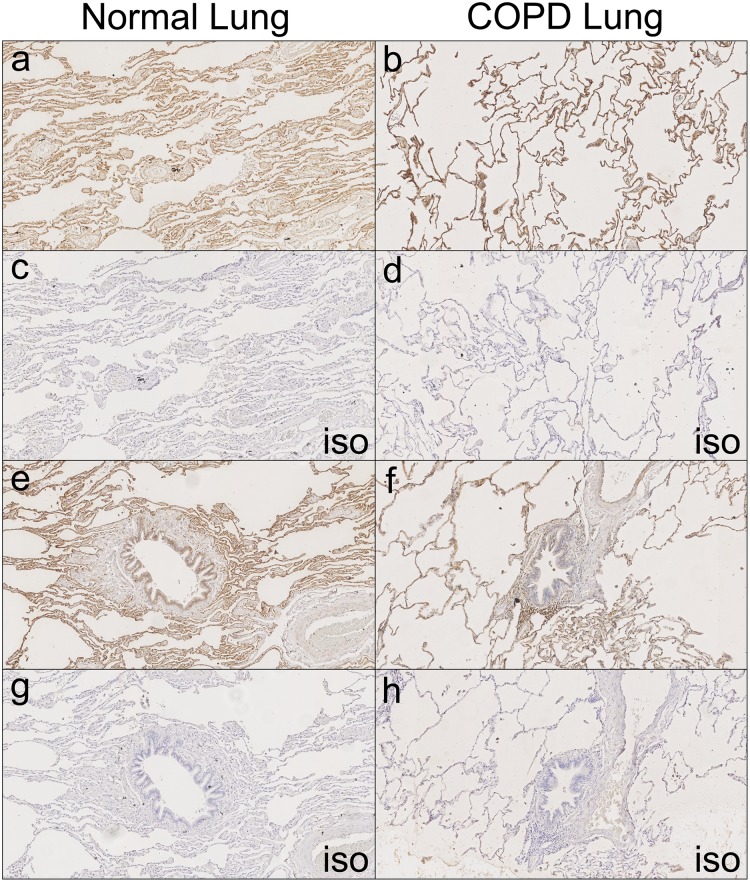
Immunohistochemical analysis of RAGE expression in healthy and COPD lung. In healthy lung tissue, RAGE was found to be localised to the cytoplasm and membrane. RAGE expression was high in the pneumocytes of alveolar regions (a). The bronchial epithelium showed variable weak to moderate staining (e). In lung tissue of individuals with COPD, RAGE was very strongly immunopositive in the membrane and cytoplasm of the pneumocytes in the alveolar regions (b). The bronchial epithelium from individuals with COPD was weak or immunonegative for the RAGE protein (f). All isotype controls were negative (c, d, g and h). Representative images of one healthy and one COPD lung shown. x10 magnification.

The expression of RAGE (encoded by *AGER*) was determined in the alveoli and bronchi of control and COPD lungs using immunohistochemistry. Specific staining was observed in both the cytoplasm and membrane (Fig 1). Pneumocytes within the alveolar regions of all three lung samples from control individuals exhibited strong staining for RAGE (Fig 1a) and in these samples the bronchial epithelium showed a variable, weaker staining pattern for RAGE (Fig 1e). In the lung tissue of all three individuals with COPD, strong immuno-positivity for RAGE was found both in the membrane and cytoplasm of pneumoctyes in the alveolar regions (Fig 1b), whilst the bronchial epithelium showed limited RAGE expression (Fig 1f).

### AGER mRNA and RAGE protein expression increase during lung development

**Fig 2:**
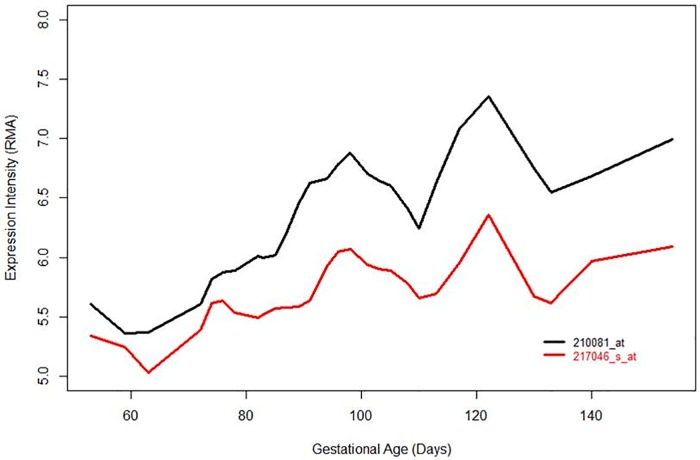
Gene expression array data of 38 fetal lungs for the *AGER* probes. Expression intensities for the *AGER* probes 210081_at and 217046_s_at were plotted against the gestational age of each fetal lung sample and showed an increase in expression with fetal lung age. RMA; Robust Multi-array Average. Affymetrix U133 Plus 2 expression array probe 210081_at probes for exon 8 of AGER mRNA whilst 217046_s_at probes AGER exons 7–11’.

The abundance of *AGER* mRNA in thirty eight human fetal lung samples was assessed using publically available expression microarray data (see methods). Levels of *AGER* mRNA were found to increase with gestational age across the Pseudoglandular and into the Canalicular stages of human lung development (gestational ages, 7–16 weeks and 17–26 weeks, respectively), (Fig 2 and Table 1).

**Table 1:** Fetal lung gene array data for *AGER* expression across Pseudoglandular and Canalicular stages.

Probe ID	AveExpr	t	*P* value	Adj *P* value	Beta-coeffient
217046_s_at	5.6868	2.985	0.0051	0.0251	0.0066
210081_at	6.3418	5.0807	9.51E-06	0.0002	0.0151

38 human lung tissue samples from gestational ages 7–22 weeks (or 53–154 days post conception) were assessed. AveExpr = average expression between all samples, t = t-statistic describing differential expression, *P* value = unadjusted *P* value, Adj.*P*.value = adjusted *P* value controlling for false discovery rate, Beta-coefficient = log (odds ratio) corresponding to the mean change in gene expression per day during the gestational age period studied, 7–22 weeks of gestational age (data analysed as described [18]).

**Fig 3:**
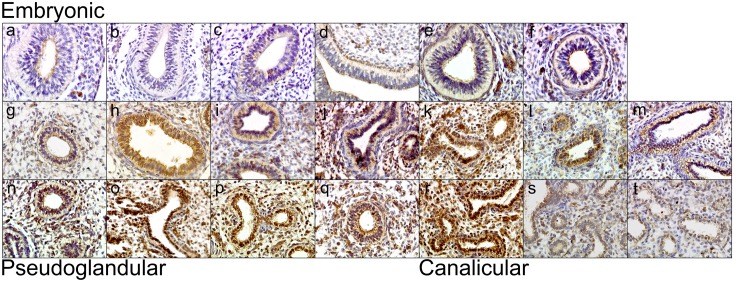
Immunohistochemical analysis of RAGE’s expression in fetal lung. Fetal lung tissue samples from embryonic (3–7 weeks), pseudoglandular (7–15 weeks) and canalicular (16–26 weeks) stages of development showed an increase in RAGE protein expression with increasing fetal lung age. a-t represent age ordered lung samples of 19 days (a, b), 21 days (c, d), 23 days (e, f), 9 weeks (g), 10 weeks (h,i,j), 11 weeks (k), 12 weeks (l,m,n), 13 weeks (o), 14 weeks (p), 15 weeks (q), 16 weeks (r), 17 weeks (s) and 19 weeks (t). All isotype controls were negative (data not shown). x40 magnification.

Having observed increasing levels of *AGER* mRNA through fetal development, levels of RAGE protein during lung development were assessed via immunohistochemistry. RAGE expression was also observed to increase during lung development across the 20 fetal lung samples; from absent to low expression in the early embryonic stages to increasing RAGE expression in the developing bronchi/bronchioles of the later embryonic stages (Fig 3a–3f). RAGE expression continued to increase across the Pseudoglandular and Canalicular stages (Fig 3g–3t).

### *AGER* mRNA levels and splice variation in airway structural cells

**Fig 4:**
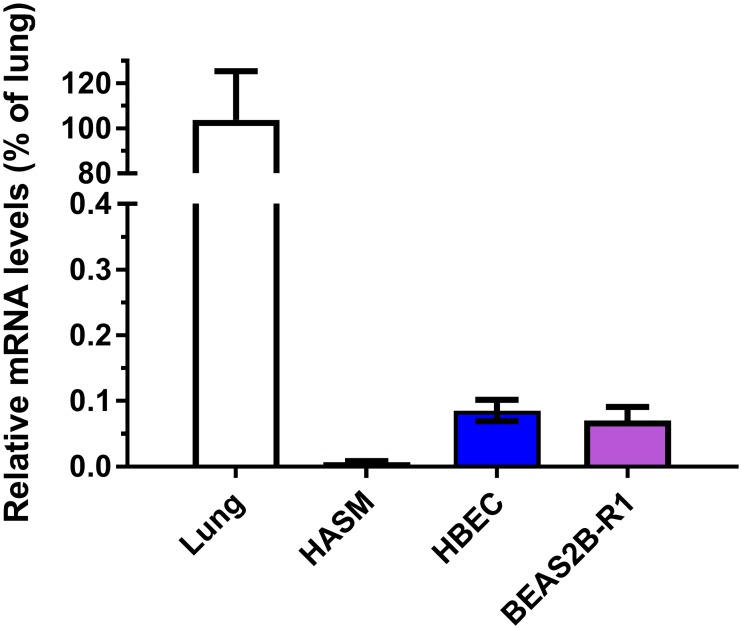
*AGER* mRNA expression levels in total lung and airway cells. Q-PCR analysis identified *AGER* mRNA was highly expressed in total lung and at lower levels in human airway smooth muscle cells (HASM) cells, human bronchial epithelial cells (HBEC) and the BEAS-2BR1 bronchial epithelial cell line, (n = 3).

**Fig 5:**
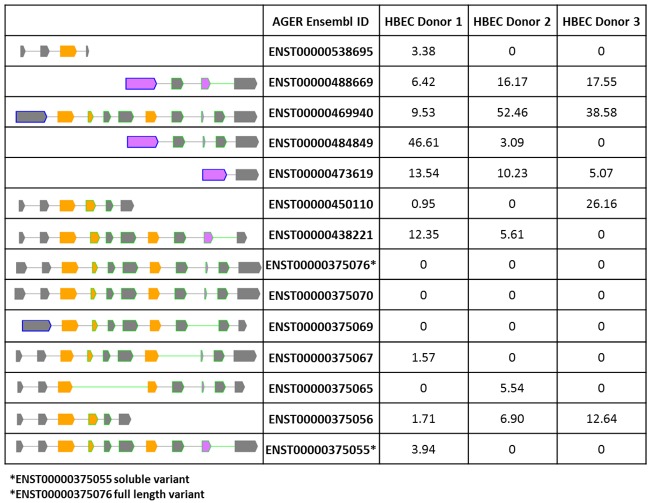
*AGER* isoform expression in three HBEC donors using RNA Seq. Structure and abundance of known *AGER* isoforms in three human bronchial epithelial cell donors illustrating heterogeneity in expression levels. Percentage abundances (% FPKM) were calculated for each donor. Transcripts for full length and soluble *AGER* were identified at similar low abundancies. FPKM; fragments per kilobase of transcript per million mapped reads.

A total *AGER* quantitative PCR (qPCR) assay was used to determine the expression of *AGER* mRNA in human lung and in airway cell populations, specifically bronchial epithelium and airway smooth muscle cells. *AGER* was highly expressed in total lung whereas cultured human airway smooth muscle, NHBEC and BEAS2B-R1 cells exhibited lower levels of *AGER* mRNA (Fig 4). Having assessed the relative abundance of *AGER* transcripts between these cell types, we then assessed which isoforms were expressed. A large number of differentially spliced *AGER* transcripts have been previously described in lung tissue [25]. To identify *AGER* transcripts in structural cells of the airways, RNA Sequencing (RNA Seq) was used to define *AGER* splicing and isoform abundance in cultured primary human bronchial epithelial cells from three independent donors (Fig 5). Using this approach we confirmed the presence and abundance of several known *AGER* transcripts in human bronchial epithelial cells, however there was heterogeneity in expression across the donors. Interestingly, several novel variants containing exons belonging to the neighbouring gene *PCL2*, spliced together with previously annotated *AGER* exons were identified. However, while spliced transcripts were seen in all three donors, there was inconsistency in the site of splicing and abundancy of novel variants and it remains unclear whether or not these transcripts are translated (data not shown).

### rs2070600 is associated with lung function and sRAGE levels in the serum of a cohort of UK smokers

We analysed 1042 DNA samples from individuals who currently smoke and have a history of smoking recruited from five UK centres to assess the association of sRAGE levels with lung function by genotype. The minor allele frequency in this population for the Ser82 variant was 10%. Serum samples were analysed from 102 of these subjects who were either homozygous for the Gly82 variant or heterozygous i.e. Gly82/Ser82. Baseline characteristics of the subjects studied are shown in Table 2.

**Table 2:** Baseline characteristics of UK smoker population used for genetic association studies.

	rs2070600 genotyping (n = 1042)	Subgroup used for serum sRAGE (Gly82Gly) (n = 51)	Subgroup used for serum sRAGE (Gly82Ser) (n = 51)
Age in years (mean ± SD)	70.0 ± 10.3 (n = 1042)	68.2 ± 7.6 (n = 51)	68.1 ± 7.9 (n = 51)
Female % (n)	43 (n = 1042)	31 (16 of 51)	31 (16 of 51)
FEV_1_ in litres (mean ± SD)	1.50 ± 0.87 (n = 1036)	1.26 ± 0.66 (n = 50)	1.30 ± 0.72 (n = 51)
FEV1 percent predicted (mean ± SD)	53.34 ± 25.47 (n = 1013)	48.00 ± 17.79 (n = 50)	48.68 ± 22.34 (n = 51)
FEV_1_/FVC as % (mean ± SD)	55 ± 17 (n = 1028)	45 ± 12 (n = 49)	48 ± 14 (n = 50)
Pack years (mean ± SD)	45 ± 28 (n = 1017)	52 ± 30 (n = 50)	52 ± 31 (n = 49)

**Fig 6:**
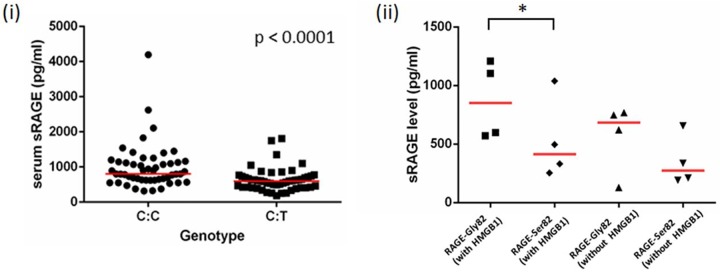
The genotype effect of rs2070600. Serum sRAGE levels are lower in individuals with the C:T genotype at rs2070600 (p<0.0001, Mann-Whitney) (6i). Cells expressing RAGE-Ser82 (T allele) stimulated with HMGB1 for 48 hours had lower levels of sRAGE than RAGE-Gly82 (C allele) carrying cells (n = 4, p = 0.036), (6ii).

We identified association between rs2070600T and increased FEV_1_ and FEV_1_/FVC ratio (*P* = 0.031 and *P* = 0.028, respectively), (S1 Table). Serum from 102 subjects in the genotyping cohort was selected based on rs2070600 genotype; providing 51 heterozygotes (C/T) and 51 homozygotes (C/C) for comparison (T/T subjects not available). These groups were matched for age, sex and smoking pack years (Table 2). Serum sRAGE levels were significantly lower in individuals with the Ser82 genotype compared to individuals homozygous for Gly82 (*P* < 0.0001) showing that that the Ser82 variant leads to reduced sRAGE levels *in vivo* (Fig 6(i) and S2 Table). We did not identify a significant association between serum sRAGE levels and FEV_1_, FVC or FEV_1_/FVC when subjects were stratified by genotype potentially due to low numbers of subjects (S3 Table).

### Activation of RAGE-Ser82 leads to lower sRAGE levels compared to RAGE-Gly82 in an epithelial cell model

To investigate the functional significance of SNP rs2070600 in the context of the full length membrane bound receptor, we created a recombinant RAGE epithelial cell model, with cells engineered to express the full length RAGE coding region with either the T allele (Ser82) or the C allele (Gly82) of rs2070600. Transient transfection of allele-specific RAGE plasmids into BEAS2B-R1 (bronchial epithelial) cells resulted in a significant induction of *AGER* mRNA (mean 2500 fold change at the mRNA level) suggesting confounding from the very low endogenous expression of RAGE is unlikely to influence our findings. Stimulation with or without HMGB1 resulted in differing levels of sRAGE secretion in a genotype specific manner (Fig 6(ii)). Cells transfected with the *AGER*-Ser82 plasmid produced lower levels of sRAGE in the presence of HMGB1 compared to RAGE-Gly82 transfected cells, *P* = 0.036 (Fig 6(ii)). We did not identify a significant difference in the levels of total *AGER* mRNA in RAGE-Ser82 versus RAGE-Gly82 expressing cells (data not shown) suggesting that the effect of the T allele of rs2070600 is not due to altered transcriptional activity.

## Discussion

A number of polymorphisms within the *AGER* locus on chromosome 6p21 have shown reproducible association with the lung function measures FEV_1_ and FEV_1_/FVC [1,2] and importantly COPD [5, 26]. The sentinel SNP at this locus is rs2070600, and the T allele at this SNP alters the amino acid sequence of RAGE from a Glycine at position 82 to a Serine, making this the most obvious candidate driving functional effects at this locus. In the current study we determined the expression profile of RAGE in healthy, diseased and the developing human lung demonstrating high protein expression in the alveoli of healthy adults and individuals with COPD. Importantly, we have shown for the first time that *AGER*/RAGE expression increases across developmental stages in human lung, both at the level of mRNA and protein, with high expression maintained in adulthood. We characterised *AGER* gene structure and transcript abundance in bronchial epithelial cells revealing many isoforms confirming the complexity of splice variation for *AGER* however we can not exclude that additional variation exists as we only investigated samples from three donors. Splice variants for *AGER* have previously been identified, with at least 10 coding for protein, however, only some are validated [25]. We confirmed that the rs2070600T allele (Ser82) is associated with higher values of FEV_1_ and FEV_1_/FVC ratio and provide new data demonstrating that this allele is associated with lower serum levels of soluble RAGE in UK smokers in keeping with findings of two previous studies [5, 27]. Levels of sRAGE have also been associated with a diagnosis of COPD, GOLD classification and several measures of emphysema using computed tomography (CT) variables [5, 28–30]. Using an allele-specific RAGE cell model we identified that activation of the RAGE-Ser82 receptor with HMGB1 ligand leads to secretion of lower levels of soluble RAGE compared to the RAGE-Gly82 receptor demonstrating that rs2070600 is functionally relevant and could underlie the effects on serum sRAGE observed *in vivo*. This effect appears to be due to altered cell processing of RAGE, as there was no obvious effect of the variants on levels of transcription.

Our immunohistochemical data confirm and extend previously published findings that showed moderate to high expression of RAGE in the alveolar region, with low expression in the bronchial epithelium [31, 32] and higher expression in lung tissue from patients with COPD[9, 10]. We observed only weak staining in bronchial epithelium. This is consistent with the low levels of mRNA found by qPCR in NHBEC and BEAS2B-R1 cells. RAGE expression has previously been shown to increase throughout development in neonatal mouse and rat lung [33, 34]. Therefore our novel finding that RAGE expression increases across human lung developmental stages both complement and extend the experiments in rodents. In keeping with the evidence presented here that RAGE expression increases with fetal age, Lopez Diez *et al*, showed high RAGE expression in 37 week old fetal lung [35]. For the *AGER* mRNA analyses across fetal stages it is important to note that using two probes to measure gene expression is a strength and based on the data of Hudson *et al*. [25] the 210081_at probe (exon 8) would measure 99% of adult lung transcripts and the 217046s probe (Exon 7–11) would measure 83% as this region spans alternative splicing forms. This is potentially reflected in the different intensities for these probes observed (Fig 4). A more recent study also suggested that the majority of *AGER* transcripts identified in both adult and fetal lung tissue contain exon 8 and so would be quantified by the 210081_at probe (Lopez-Diez 2013).

A limitation of the fetal immunohistochemistry was the low number of samples at each developmental stage (especially canalicular). The relationship between increasing RAGE expression in the fetal lung with increasing age may potentially be explained by cell differentiation and the evolving cellular composition within the developing lung. During the embryonic stage, early precursor lung cells are present and with increasing differentiation at the pseudoglandular stage, alveolar type II pneumocytes are present which express RAGE. Furthermore, at the canalicular stage of lung development type I pneumocytes are present alongside a larger differentiated population of type II pneumocytes expressing RAGE.

Importantly, a functional role for RAGE during lung development has recently been demonstrated in mice, with alterations in RAGE levels causing lethality and/or changes to secondary septation, hypoplasia, lung tissue loss and changes in pulmonary microvasculature [15, 36–38]. While we cannot evaluate these mechanisms in the human context our differential expression data in human fetal lung implies a role for *AGER* in mammalian lung development. Taken together, these data show that RAGE expression increases through lung development and that high expression is maintained in the adult lung suggesting a role for RAGE in lung homeostasis. This has implications for the mechanisms underlying the development of COPD as these finding potentially suggest that individuals with alterations in *AGER* homeostasis may have defective lung development and therefore never reach maximal lung function. This is at least in part supported by the association of *AGER* SNPs and lung function in children [2]. One limitation of the immunohistochemical study of fetal lung tissue was the availability of only a relatively small number of samples at each developmental stage prohibiting more quantitative or semi-quantitative analyses. A separate limitation of the study was that we expression profiled *AGER* mRNA expression in the BEAS2B-R1 immortalised cell line rather than in lung tissue or primary cells which would be optimal.

Overall, our data suggest that the Gly82Ser polymorphism alters ligand-dependent release of sRAGE in the airway. These data compliment and extend previous reports in non-airway cell systems where it has been shown that Gly82Ser can influence ligand binding and/or receptor activation [12, 39, 40]. RAGE-Ser82 receptors expressed on CHO [12] or COS7 [39] cells have a lower dissociation constant (K_d_) for the RAGE ligand EN-RAGE or glycolaldehyde-derived AGE respectively compared to RAGE-Gly82 receptors. This reduced K_d_ for glycolaldehyde-derived AGE for RAGE-Ser82 receptors resulted in an enhanced VEGF mRNA induction demonstrating downstream effects [39]. Subsequently, Park and colleagues demonstrated that RAGE-Ser82 was consistently glycosylated at Asn81 *in vitro* whereas RAGE-Gly82 was not [40]. This N-glycosylation was shown to be critical for the regulation of ligand (S100B) binding with potential consequences for NFКB pathway activation in HEK293 cells expressing RAGE [40]. In our epithelial cell system the RAGE-Ser82 variant was significantly associated with decreased levels of secretion of sRAGE by the cell in response to activation with the ligand HMGB1. One limitation of the cell experiments is that we did not also measure NFКB pathway activation which would have provided greater depth to our findings. However, taken together, these data suggest that rs2070600 is likely to be the functional variant which accounts for the observed association with lung function variables as our *in vitro* model only contains this specific variant change, as opposed to other SNPs in linkage disequilibrium (LD) with rs2070600 [5].

It has been robustly demonstrated that sRAGE levels in serum or plasma of COPD patients is lower compared to control subjects and associated with GOLD stage and markers of emphysema (reviewed by Yonchuk et al 2015)[41]. This led to the concept that this lower level of sRAGE, which acts as a decoy receptor to limit inflammation may lead to greater RAGE driven inflammation in COPD. A limitation of our study is that by using samples from a general population of smokers with and without COPD we did not have data on the extent of emphysema in the individuals with COPD. Additional studies investigating the distribution of RAGE/sRAGE within the lung would help (i) clarify the source(s) of serum sRAGE (potentially alveolar epithelial cells or pulmonary capillary endothelial cells) and (ii) identify if the relative contribution of these sources is altered in COPD.

The relationship between *AGER* genetics, lung function and sRAGE levels is potentially counterintuitive as the rs2070600T allele is associated with better lung function in the general population and lower sRAGE. The molecular mechanisms underlying these observations still remain to be defined, however our novel data provide supporting evidence that rs2070600 is the key SNP driving sRAGE levels in the cell assays. The key SNP(s) driving the association with lung function and COPD diagnosis at the population level remain to be elucidated and similarly whether rs2070600 contributes to lung function and sRAGE via overlapping or distinct mechanisms is an important, unresolved question.

In summary, we provide important new information regarding the expression profile of RAGE in the human lung by identifying the isoforms present, and by assessing differential expression in human fetal lung development and in human airways from control individuals and those with COPD. The T allele of rs2070600, which codes for the RAGE-Ser82 variant, was found to be associated with increased lung function in a cohort of UK smokers and the levels of sRAGE were found to be lower in the serum of smoking individuals carrying this variant. These data provide novel insight into the role of *AGER*/RAGE in lung biology and support a functional role for rs2070600 in determining sRAGE levels, which may in turn explain the association seen between this variant and function.

## Supporting Information

S1 Table Linear Regression analysis of lung function measures and rs2070600 genotyping. rs2070600T was associated with increased FEV_1_ and FEV_1_/FVC ratio. Minor allele frequency of tested population = 10%. Coded allele = T.
(PDF)

S2 Table Serum soluble RAGE levels in 102 individual stratified by rs2070600 genotype. Serum sRAGE levels were lower in individuals with a C:T genotype for rs2070600.
(PDF)

S3 Table Linear regression analysis of serum sRAGE level and lung function measures FEV1, FVC and FEV1/FVC. Serum sRAGE levels was not associated with FEV_1_, FVC or FEV_1_/FVC when subjects were stratified by genotype. F = statistical significance of the regression equation as a whole.
(PDF)
