# Supplementary material for: The Ser82 RAGE Variant Affects Lung Function and Serum RAGE in Smokers and sRAGE Production In Vitro
Source: PLoS One. 2016 Oct 18;11(10):e0164041. doi: 10.1371/journal.pone.0164041 (PMC5068780; doi:10.1371/journal.pone.0164041)
Supplement: S1 Table — rs2070600T was associated with increased FEV1 and FEV1/FVC ratio. Minor allele frequency of tested population = 10%. Coded allele = T. (PDF) [file pone.0164041.s001.pdf]

Title: Characterisation of *AGER* in the airways and periphery

Supplementary File

S1 Table. Linear Regression analysis of lung function measures and rs2070600 genotyping. rs2070600T was associated with increased FEV1 and FEV1/FVC ratio. Minor allele frequency of tested population = 10%. Coded allele = T.

1a: FEV_1

FEV_1 β	FEV_1 SE	FEV_1 P value
0.145	0.067	0.031

1b: FVC

FVC β	FVC SE	FVC P value
0.086	0.072	0.23

1c: FEV_1/FVC

FEV_1/FVC β	FEV_1/FVC SE	FEV_1/FVC P value
0.033	0.015	0.028
