# Supplementary material for: The Ser82 RAGE Variant Affects Lung Function and Serum RAGE in Smokers and sRAGE Production In Vitro
Source: PLoS One. 2016 Oct 18;11(10):e0164041. doi: 10.1371/journal.pone.0164041 (PMC5068780; doi:10.1371/journal.pone.0164041)
Supplement: S2 Table — Serum sRAGE levels were lower in individuals with a C:T genotype for rs2070600. (PDF) [file pone.0164041.s002.pdf]

Title: Characterisation of *AGER* in the airways and periphery

Supplementary File

S2 Table. Serum soluble RAGE levels in 102 individual stratified by rs2070600 genotype.
Serum sRAGE levels were lower in individuals with a C:T genotype for rs2070600.

rs2070600 genotype	sRAGE (pg/ml)	Mean	Median	Samples
C:C	319 - 4197	993	801	51
C:T	183 - 1811	633	597	51
