# Supplementary material for: The Ser82 RAGE Variant Affects Lung Function and Serum RAGE in Smokers and sRAGE Production In Vitro
Source: PLoS One. 2016 Oct 18;11(10):e0164041. doi: 10.1371/journal.pone.0164041 (PMC5068780; doi:10.1371/journal.pone.0164041)
Supplement: S3 Table — Serum sRAGE levels was not associated with FEV1, FVC or FEV1/FVC when subjects were stratified by genotype. F = statistical significance of the regression equation as a whole. (PDF) [file pone.0164041.s003.pdf]

Title: Characterisation of *AGER* in the airways and periphery

Supplementary File

S3 Table. Linear regression analysis of serum sRAGE level and lung function measures FEV1, FVC and FEV1/FVC. Serum sRAGE levels was not associated with FEV_1, FVC or FEV_1/FVC when subjects were stratified by genotype. F = statistical significance of the regression equation as a whole.

Samples	Lung function measure	F	P value
All (n = 102)	FEV_1	0.869	0.354
	FVC	0.379	0.504
	FEV_1/FVC	0.515	0.475
C:C genotype	FEV_1	0.493	0.486
	FVC	1.195	0.280
	FEV_1/FVC	0.385	0.535
C:T genotype	FEV_1	0.063	0.804
	FVC	0.001	0.982
	FEV_1/FVC	0.002	0.968
